# Decreased telomere length in a subgroup of young individuals with bipolar disorders: replication in the FACE-BD cohort and association with the shelterin component POT1

**DOI:** 10.1038/s41398-024-02824-z

**Published:** 2024-03-01

**Authors:** Luana Spano, Cynthia Marie-Claire, Ophélia Godin, Apolline Lebras, Cindie Courtin, Jean-Louis Laplanche, Marion Leboyer, Bruno Aouizerate, Antoine Lefrere, Raoul Belzeaux, Philippe Courtet, Emilie Olié, Caroline Dubertret, Raymund Schwan, Valérie Aubin, Paul Roux, Mircea Polosan, Ludovic Samalin, Emmanuel Haffen, Fondamental Advanced Centers Of Expertise In Bipolar Disorders (Face-Bd) Collaborators, B. Etain, E. Olié, M. Leboyer, E. Haffen, P. M. Llorca, V. Barteau, S. Bensalem, O. Godin, H. Laouamri, K. Souryis, S. Hotier, A. Pelletier, N. Drancourt, J. P. Sanchez, E. Saliou, C. Hebbache, J. Petrucci, L. Willaume, E. Bourdin, F. Bellivier, B. Etain, V. Hennion, E. Marlinge, P. Lebard, B. Antoniol, A. Desage, S. Gard, A. Jutant, K. Mbailara, I. Minois, L. Zanouy, C. Abettan, L. Bardin, A. Cazals, P. Courtet, B. Deffinis, D. Ducasse, M. Gachet, A. Henrion, E. Martinerie, F. Molière, B. Noisette, E. Olié, G. Tarquini, J. M. Azorin, R. Belzeaux, N. Correard, J. L. Consoloni, F. Groppi, L. Lescalier, J. Montant, M. Rebattu, N. Viglianese, R. Cohen, J. P. Kahn, M. Milazzo, O. Wajsbrot-Elgrabli, T. Bougerol, B. Fredembach, Q. Denoual, A. Bertrand, A. Pouchon, M. Polosan, L. Brehon, G. Bony, L. Durand, V. Feuga, N. Kayser, C. Passerieux, P. Roux, V. Aubin, I. Cussac, M. A. Dupont, J. Loftus, I. Medecin, C. Dubertret, N. Mazer, C. Portalier, C. Scognamiglio, A. Bing, P. Laurent, C. Beal, O. Blanc, T. Bonnet, D. Lacelle, P. M. Llorca, M. Mennetrier, L. Samalin, M. Vayssié, Frank Bellivier, Bruno Etain

**Affiliations:** 1 Université Paris Cité, INSERM UMR-S 1144, Optimisation Thérapeutique en Neuropsychopharmacologie OTeN, Paris, France; 2 Fondation FondaMental, Créteil, France; 3 Université Paris Est Créteil, INSERM U955, IMRB, Translational NeuroPsychiatry Laboratory, Créteil, France; 4 Département de Biochimie et Biologie Moléculaire, DMU BioGeM, Hôpitaux Lariboisière-Fernand Widal, GHU APHP.Nord – Université de Paris, Paris, France; 5 AP-HP, Hôpitaux Universitaires Henri Mondor, Département Médico-Universitaire de Psychiatrie et d’Addictologie (DMUIMPACT), Fédération Hospitalo-Universitaire de Médecine de Précision en Psychiatrie (FHU ADAPT), Créteil, France; 6 Centre Hospitalier Charles Perrens, Laboratoire NutriNeuro (UMR INRA 1286), Université de Bordeaux, Bordeaux, France; 7 Pôle de Psychiatrie, Assistance Publique Hôpitaux de Marseille, Marseille, France; 8 INT-UMR7289, CNRS Aix-Marseille Université, Marseille, France; 9 Université Montpellier, Montpellier, France; 10 Department of Emergency Psychiatry and Acute Care, CHU Montpellier, IGF, Univ. Montpellier, CNRS, INSERM, Montpellier, France; 11 AP-HP, Groupe Hospitalo-Universitaire AP-HP Nord, DMU ESPRIT, Service de Psychiatrie et Addictologie, Hôpital Louis Mourier, Colombes, France; 12 Université de Paris, Inserm UMR1266, Sorbonne Paris Cité, Faculté de Médecine, Paris, France; 13 Université de Lorraine, Centre Psychothérapique de Nancy, Inserm U1254, Nancy, France; 14 Pôle de Psychiatrie, Centre Hospitalier Princesse Grace, Monaco, Monaco; 15 Centre Hospitalier de Versailles, Service Universitaire de Psychiatrie d’Adulte et d’Addictologie, Le Chesnay, France; 16 Equipe DisAP-PsyDev, CESP, Université Versailles Saint- Quentin-en-Yvelines – Paris-Saclay, Inserm, Villejuif, France; 17 Université Grenoble Alpes, Inserm, U1216, CHU Grenoble Alpes, Grenoble Institut Neurosciences, Grenoble, France; 18 Centre Hospitalier et Universitaire, Département de Psychiatrie, Université Clermont Auvergne, CNRS, Clermont Auvergne INP, Institut Pascal (UMR 6602), Clermont-Ferrand, France; 19 Service de Psychiatrie de l’Adultre, CIC-1431 INSERM, CHU de Besançon, Laboratoire de Neurosciences, UFC, UBFC, Besançon, France; 20 AP-HP, Groupe Hospitalo-Universitaire AP-HP Nord, DMU Neurosciences, Hôpital Fernand Widal, Département de Psychiatrie et de Médecine Addictologique, Paris, France; 21 FACE-BD Clinical Coordinating Center (Fondation FondaMental), Créteil, France; 22 FACE-BD Data Coordinating Center (Fondation FondaMental), Créteil, France; 23 FACE-BD Clinical Sites and Principal Collaborators in France, Montpellier, France; 24 AP-HP, DHU PePSY, Pôle de Psychiatrie et d’Addictologie des Hôpitaux Universitaires H Mondor, Créteil, France; 25 AP-HP, GH Saint-Louis–Lariboisière–Fernand Widal, Pôle Neurosciences, Paris, France; 26 Hôpital C. Perrens, Centre Expert Trouble Bipolaire, Service de Psychiatrie Adulte, Pôle 3-4-7, Bordeaux, France; 27 Département d’Urgence et Post Urgence Psychiatrique, CHRU Montpellier, Montpellier, France; 28 Pôle de Psychiatrie, addictologie et pédopsychiatrie, Hôpital Sainte Marguerite, Marseille, France; 29 Service de Psychiatrie et Psychologie Clinique, CHU de Nancy, Hôpitaux de Brabois, Vandoeuvre Les Nancy, France; 30 Service Universitaire de Psychiatrie, CHU de Grenoble et des Alpes, Grenoble, France; 31 Centre Hospitalier de Versailles, Service Universitaire de Psychiatrie d’adultes, Le Chesnay, France; 32 Centre Hospitalier Princesse Grace, Monaco, Monaco; 33 APHP, Groupe Hospitalo-universitaire AP-HP Nord, DMU ESPRIT, Service de Psychiatrie et Addictologie, Hôpital Louis Mourier, Colombes, France; 34 Service de Psychiatrie d’adultes B, Centre Expert Trouble Bipolaire, CHU de Clermont-Ferrand, Clermont-Ferrand, France

**Keywords:** Bipolar disorder, Biomarkers

## Abstract

Bipolar disorder (BD) has been associated with premature cellular aging with shortened telomere length (TL) as compared to the general population. We recently identified a subgroup of young individuals with prematurely shortened TL. The aims of the present study were to replicate this observation in a larger sample and analyze the expression levels of genes associated with age or TL in a subsample of these individuals. TL was measured on peripheral blood DNA using quantitative polymerase chain reaction in a sample of 542 individuals with BD and clustering analyses were performed. Gene expression level of 29 genes, associated with aging or with telomere maintenance, was analyzed in RNA samples from a subsample of 129 individuals. Clustering analyses identified a group of young individuals (mean age 29.64 years), with shorter TL. None of the tested clinical variables were significantly associated with this subgroup. Gene expression level analyses showed significant downregulation of *MYC*, *POT1*, and *CD27* in the prematurely aged young individuals compared to the young individuals with longer TL. After adjustment only *POT1* remained significantly differentially expressed between the two groups of young individuals. This study confirms the existence of a subgroup of young individuals with BD with shortened TL. The observed decrease of *POT1* expression level suggests a newly described cellular mechanism in individuals with BD, that may contribute to telomere shortening.

## Introduction

A 10–15 years decrease in life expectancy has been repeatedly observed in individuals with bipolar disorder (BD) as compared to the general population [1, 2]. Individuals with BD experience higher rates of comorbidities associated with aging, such as type II diabetes, metabolic syndrome, cancer, immune dysregulation, cardiovascular and cerebrovascular disorders [3–5]. These observations suggest that BD may be associated with a premature cellular aging [6], which can be estimate by various molecular markers [7].

Telomere length (TL) is the most well-described biomarker of cellular aging. Telomeres are repeated sequences of non-coding nucleotides (TTAGGG). Since they are located at the end of chromosomes, they protect chromosomes from their degradation and fusion [8]. However, at each cell division, the replication of DNA is incomplete, leading to telomere shortening with aging [9]. Several mechanisms are at stake to preserve TL, such as the “shelterin complex” which involves the interaction between six proteins and the telomerase, an enzyme that adds TTAGGG sequences to the telomeres [9]. TL, as a marker of aging, has been shown to be decreased in individuals with BD compared to the unaffected or healthy population in a meta-analysis [10]. Moreover, in BD, we have recently identified a subgroup of individuals in their thirties but with a TL that was as short as the one of those individuals in their sixties [11]. However, it remains unclear whether this decrease of TL observed in individuals with BD is associated with specific clinical characteristic, even if some potential determinants have been proposed [12]. For example, long-term lithium treatment would have a positive effect on telomere attrition [13] while childhood maltreatment [14], as well as a higher number of mood episodes [15], would have a negative impact on TL. Some alterations of circadian rhythms (i.e. circadian misalignment) might also contribute to shorten telomeres, as we suggested in a recent article [16].

The identification of genes whose expression levels change with age and, independently of chronological age, predict accelerated decline of health is an expanding area of research [17]. A transcriptomic age predictor has been proposed using a whole-blood gene expression meta-analysis in 14,983 individuals of European ancestry where 1,497 genes were identified as differently expressed with chronological age [18].

Among these age-related genes, those involved in telomere structure and maintenance have been also recently reviewed [19] and their expression levels studied in the general population. For example, a recent study of the data from a whole genome sequencing of 250 Dutch family trios analyzed TL inheritance patterns and associated genetic factors. *RRM1*, a gene playing a role in cellular proliferation, was suggested to be involved in TL regulation [20]. To our knowledge, no previous study has analyzed simultaneously the expression of age and TL-related genes in individuals with BD. Such a study in individuals with BD would help identifying potential molecular determinants of telomere shortening in this population.

In this study, we first analyzed TL in individuals with BD to replicate, in a larger cohort and with a larger set of clinical variables, the identification of a sub-group of young individuals with telomere shortening. Second, we analyzed expression levels of genes associated with age or TL in a subsample of these individuals.

## Materials and methods

### Participants

542 individuals from the multicenter cross-sectional study, FACE-BD (FondaMental Advanced Center of Expertise for Bipolar Disorders), were included in this study. All participants were aged 16 years or older and were diagnosed with BD type I, type II or NOS (not otherwise specified) according to DSM-IV criteria (Diagnostic and Statistical Manual of Mental Disorders - fourth edition) [21]. This cohort has been previously described in details [22]. Individual were selected for having the most completed data collected on age, sex, Montgomery Asberg Depression Scale (MADRS) [23], Young Mania Rating Scale (YMRS) [24], number of mood episodes, age of onset, Body mass Index (BMI), tobacco and childhood traumatic events (assessed using the Childhood Trauma Questionnnaire, CTQ) [25]. They also completed questionnaires to assess their sleep quality and rhythm with the Pittsburgh Sleep Quality Index (PSQI) [26] and the Composite Scale of Morningness (CSM) [27]. The assessment protocol was approved by the institutional review board (Comité de Protection des Personnes Ile de France IX; January 18, 2010), in accordance with the French laws for non-interventional studies and requires only an informational letter.

### qPCR for TL measurements

A blood sample was collected at inclusion for some of the individuals who participated to the BioPsy or PsyCoh study (ANR-11-IDEX-0004-02 and ANR-10-COHO-10-01). A specific informed consent form was signed by all participants to have an additional blood sample for research purpose. DNA was extracted from peripheral blood samples of 542 patients. TL was analyzed using real-time quantitative polymerase chain reaction (qPCR) carried out on a CFX384 Touch Real-Time PCR Detection System (Biorad), as previously described [11]. To summarize, Beta-hemoglobin was used as a single-copy standard for a relative quantification of TL and all samples were assessed in triplicate. qPCRs were performed in 10 μL of reaction containing 25 ng of DNA, 300 nmol/L of each primer and 5 μL of 2× SsoAdvanced Universal SYBR Green Supermix (Biorad). T/S ratio was obtained using the delta delta Cq calculation method.

### Gene expression measurements

To measure gene expression levels, we performed RT-qPCR (reverse transcriptase – qPCR) in 129 individuals. These samples were selected on the basis of the availability of RNA Sample. Total RNA was isolated using a Maxwell 16 LEV simply RNA Purification Kit (Promega, France). For the 129 individuals, 200 ng of RNA were reverse transcribed in a final volume of 60 µL using the iScript Reverse Transcription Supermix (Bio-Rad Laboratories, France). Following the manufacturer’s protocol, the reaction mix was incubated 5 min at 25 °C, 20 min at 46 °C and 1 min at 95 °C. At the end of the reaction, cDNA samples were stored at −20 °C. Based on their expression levels in blood from the GTEX portal (https://gtexportal.org/home/), 40 genes of interest implicated in the homeostasis of TL [19, 20] or whose expression levels have been previously associated with age [18, 28, 29] were selected (Supplementary 1). Six reference genes *B2M*, *HPRT1*, *HSP90AB1*, *RPL30*, *RPS18*, and *SDHA* were chosen based on previous data from our group [30] (Supplementary 1). Custom designed 384 wells Prime PCR plates were preplated (Bio-Rad laboratories, France). Quantitative PCR were performed following the manufacturer’s protocol for PrimePCR Assays (Bio-Rad laboratories, France) in a final volume of 10 µL with 2X of SsoAdvanced universal Supermix (Bio-Rad laboratories, France) and 0.5 µL of cDNA sample. The thermal cycling conditions were as follow: polymerase activation step at 95 °C for 2 min followed by 40 cycles of 95 °C for 5 sec and 60 °C for 30 s for denaturation and annealing/extension step. At the end of amplification, a melting curve was used to verify the specificity of primers. These amplifications were carried out on a CFX384 Touch Real-Time PCR Detection System (Biorad laboratories, France). Delta delta Cq calculation method was used to obtain relative level of gene expression. All samples were assessed in duplicate. For eleven genes (*CCDC88C*, *CHAF1A*, *CHAF1B*, *CRP*, *DCLRE1B*, *EXO1*, *EZH2*, *TERF2*, *TERT*, *TP53*, and *WRN*), the RT-qPCR results did not meet the quality control criteria of the MIQE guidelines (Cq > 30 or multiple peaks melting curve) [31]. Therefore, a total of 29 genes could be analyzed. The built-in GeNorm algorithm, in CFX Maestro Software (Bio-Rad laboratories, France) was used to calculate normalization factor from the six reference genes.

### Statistical analysis

Statistical analysis were performed on JASP (Version 0.14.1), JAMOVI (Version 1.6.23.0), Prism (Version 9), and Rstudio 2 (Version 1.2.1335) softwares. When *p*-values were < 0.05, results were considered significant.

A k-means clustering analysis was used to classify the 542 subjects using age and TL (standardized values) as classification variables with the following options : maximal number of clusters set at 10, means used as center type and Lloyd-Forgy algorithm. The elbow method was used to determine the optimal number of cluster. Kruskal-Wallis followed by pairwise comparisons Dunn’s tests for continuous variables (clinical variables and levels of expression) and Chi2 tests for categorical variables were performed to compare the clusters. A logistic regression was used to test for the association between the genes found significantly different between the two youngest clusters with adjustments for age, sex, BMI and tobacco. Spearman’s correlations were used to investigate correlation between genes and TL in between the two youngest clusters. In this exploratory study of gene expression no correction for multiple comparison was made.

## Results

### Sample characteristics

Socio-demographic and clinical characteristics of the sample (*N* = 542) are presented in Supplementary 2. In summary, the median age of the sample was 33 years old and there were more women than men (64.4%). BD type 1 was diagnosed among 46.4% of the participants. Almost half of the individuals (47.44%) were current tobacco smokers and a quarter (25.74%) had a past lifetime alcohol misuse. More than a third of the participants were currently treated with lithium (39.44%) and antidepressants (ATD) (34.06%). Almost half of them were currently treated with atypical antipsychotic agents (APA) (44.69%) or with anticonvulsant agents (ACA) (50%). The median childhood trauma score of this sample was 38 and the median relative TL was 2.36. TL was negatively correlated with age in the sample (Spearman’s rho = -0.236, *p* < 0.001).

### Clustering analysis with age and TL

**Fig. 1:**
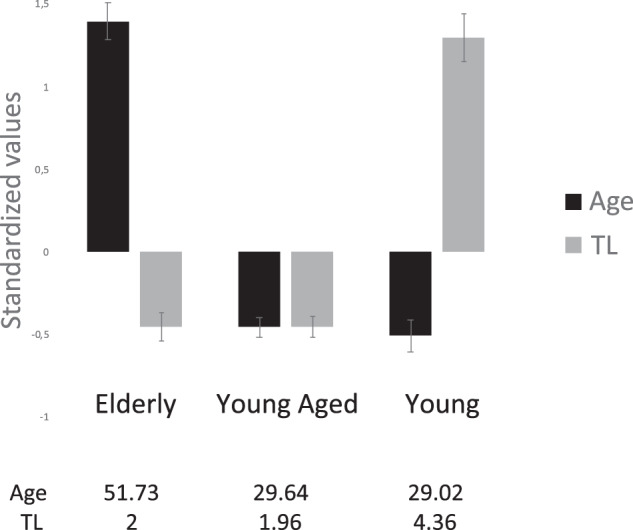
Clustering of 542 BD patients with age and TL as classification variables. Standardized values of age and TL were used to create clusters.

The elbow method identified three subgroups of individuals (Supplementary 3a). As shown in Fig. 1, the first cluster named “Elderly” was a subgroup with an older age and a decreased TL (*N* = 138 ; mean age = 51.73 ; mean TL = 2). The second named “Young Aged” consisted of individuals with a young age but with a similar TL as the oldest cluster (N = 265 ; mean of age = 29.64 ; mean of TL = 1.96). The last subgroup named “Young” consisted in young individuals with preserved TL (*N* = 139 ; mean of age = 29.02 ; mean of TL = 4.36). A graph with the clusters of points resulting from this k-means clustering analysis is shown in Supplementary 3b

These three clusters were compared for all available clinical variables. Results are shown in Table 1. Most differences are found between the “Elderly” cluster and the two other clusters (i.e. youngest individuals). Individuals in “Elderly” cluster had higher BMI (*p* = 0.039), age of onset (*p* < 0.001), CSM score (*p* < 0.001) and total number of medications (*p* = 0.013), as compared to the two youngest clusters. This “Elderly” cluster also had fewest current smokers (*p* < 0.001) and cannabis users (*p* < 0.001), as compared to the two youngest clusters.

**Table 1:** Comparisons between the three clusters for clinical variables.

Variables	C1 Elderly (*N* = 138)	C2 Young Aged (*N* = 265)	C3 Young (*N* = 139)	*p* value global	Pairwise comparisons
Age (years), mean ± SD	51.73 ± 7.97	29.64 ± 6.11	29.02 ± 7.09	*<0.001*^*a*^	1 > 2 = 3 (*p* < 0.001^c^)
TL, mean ± SD	2.00 ± 0.86	1.96 ± 0.71	4.36 ± 1.19	*<0.001*^*a*^	1 = 2 < 3 (*p* < 0.001^c^)
Sex (Female), %	88 (63.8%)	171 (64.5%)	90 (64,7%)	0.984^b^	
BMI (kg/m^2^), mean ± SD	26.58 ± 5.78	25.36 ± 5.12	24.92 ± 5.19	*0.039*^*a*^	1 > 3 (*p* = 0.014^c^) 1 = 2 (*p* = 0.050^c^) 2 = 3 (*p* = 0.384^c^)
Tobacco current smoker (yes), %	40 (29.0%)	127 (47.9%)	74 (53.2%)	*<0.001*^*b*^	1 < 2 = 3 (*p* < 0.001^b^)
Past lifetime alcohol misuse (yes), %	26 (18.8%)	66 (24.9%)	29 (20.9%)	0.384^b^	
Past lifetime cannabis misuse (yes), %	14 (10.1%)	72 (27.2%)	32 (23.0%)	*<0.001*^*b*^	1 < 2 (*p* < 0.001^b^) 1 < 3 (*p* = 0.002^b^) 2 = 3 (*p* = 0.493^b^)
MADRS, mean ± SD	9.59 ± 8.23	10.73 ± 9.51	10.64 ± 9.50	0.767^a^	
YMRS, mean ± SD	2.53 ± 3.64	2.22 ± 3.51	1.93 ± 2.66	0.543^a^	
BD type 1 (yes), %	62 (44.9%)	115 (43.4%)	74 (53.2%)	0.158^b^	
Age of onset (years), mean ± SD	27.77 ± 11.12	19.90 ± 5.75	19.48 ± 4.94	*<0.001*^*a*^	1 > 2 = 3 (*p* < 0.001^c^)
Duration of illness (years), mean ± SD	23.97 ± 11.09	9.77 ± 5.99	9.56 ± 6.48	*<0.001*^*a*^	1 > 2 = 3 (*p* < 0.001^c^)
Number of mood episodes, mean ± SD	10.72 ± 9.90	7.31 ± 7.17	7.12 ± 7.22	*<0.001*^*a*^	1 > 2 = 3 (*p* < 0.001^c^)
CTQ total score, mean ± SD	42.98 ± 14.72	40.72 ± 13.25	41.53 ± 14.73	0.237^a^	
Lithium (yes), %	56 (45.2%)	67 (34.2%)	47 (42.3%)	0.113^b^	
ACA (yes), %	68 (55.3%)	101 (50.8%)	48 (42.9%)	0.157^b^	
APA (yes), %	50 (36.2%)	85 (32.1%)	50 (36.0%)	0.716^b^	
ATD (yes), %	45 (32.6%)	63 (23.8%)	33 (23.7%)	0.566^b^	
Total number of medictions, mean ± SD	2.16 ± 1.14	1.87 ± 1.03	1.82 ± 0.96	*0.013*^*a*^	1 > 2 = 3 (*p* = 0.01^c^)
PSQI, mean ± SD	6.75 ± 3.90	7.00 ± 3.45	6.61 ± 3.38	0.398^a^	
CSM, mean ± SD	37.94 ± 7.47	32.94 ± 7.41	32.60 ± 7.10	*<0.001*^*a*^	1 > 2 = 3 (*p* < 0.001^c^)

*TL* Telomere Length, *BMI* Body Mass Index, *MADRS* Montgomery Asberg Depression Rating Scale, *YMRS* Young Mania Rating Scale, *BD* Bipolar Disorder, *CTQ* Childhood Trauma Questionnaire, *AC* Anticonvulsant agent, *APA* Atypical Antipsychotic agent, *ATD* Antidepressant, *PSQI* Pittsburgh Sleep Quality Index, *CSM* Composite Scale of Morningness.

^a^Kruskal-Wallis test; ^b^chi2 test; ^c^Dunn’s test.

Individuals in “Young Aged” and “Young” clusters were similar in age, but differed only for their TL. Therefore, we did not identify any clinical variable that differentiated these 2 clusters.

### Associations between age-related genes and clusters

Gene expression levels of the 29 age-related genes were compared using Kruskal-Wallis analysis between the 3 clusters (Supplementary 4) and only genes that differed at a *p*-value threshold of 0.05 are presented in Table 2. Expression levels of 8 genes were found to be different for this threshold between the three clusters: *CD27*, *LDHB*, *LEF1*, *LTB*, *MYC*, *PIK3IP1*, *POT1*, and *RPS6*. For seven of the 8 genes (except *LTB*), “Young Aged” cluster had mean values of expression that were numerically between values observed for the “Young” cluster and for the “Elderly” cluster.

**Table 2:** Comparisons of genes expression between the three clusters.

Genes	C1 Elderly mean ± SD (*n* = 16)	C2 Young aged mean ± SD (*n* = 68)	C3 Young mean ± SD (*n* = 45)	Statistic	*p* value^a^	*Pairwise comparisons*^*b*^
*CD27*	0.19 ± 0.07	0.34 ± 0.29	0.43 ± 0.46	11.74	*0.003*	*1* < *2 (p* = *0.04)* *1* < *3 (p* < *0.001)* ***2*** < ***3 (p*** = ***0.038)***
*LDHB*	1.28 ± 0.71	1.63 ± 0.64	1.80 ± 0.68	7.11	*0.029*	*1* = *2 (p* = *0.063)* *1* < *3 (p* = *0.008)* *2* = *3 (p* = *0.19)*
*LEF1*	0.31 ± 0.16	0.51 ± 0.48	0.60 ± 0.48	7.63	*0.022*	*1* = *2 (p* = *0.118)* *1* < *3 (p* = *0.008)* *2* = *3 (p* = *0.079)*
*LTB*	0.52 ± 0.18	0.76 ± 0.42	0.74 ± 0.33	8.63	*0.013*	*1* < *2 (p* = *0.005)* *1* < *3 (p* = *0.006)* *2* = *3 (p* = *0.92)*
*MYC*	0.11 ± 0.05	0.20 ± 0.24	0.30 ± 0.43	10.62	*0.005*	*1* = *2 (p* = *0.091)* *1* < *3 (p* = *0.002)* ***2*** < ***3 (p*** = ***0.027)***
*PIK3IP1*	0.20 ± 0.10	0.30 ± 0.27	0.38 ± 0.39	7.01	*0.030*	*1* = *2 (p* = *0.230)* *1* < *3 (p* = *0.015)* *2* = *3 (p* = *0.053)*
*POT1*	1.26 ± 0.73	1.40 ± 0.66	1.67 ± 0.68	6.43	*0.040*	*1* = *2 (p* = *0.57)* *1* < *3 (p* = *0.045)* ***2*** < ***3 (p*** = ***0.027)***
*RPS6*	1.20 ± 0.45	1.48 ± 0.44	1.53 ± 0.41	6.60	*0.037*	*1* < *2 (p* = *0.046)* *1* < *3 (p* = *0.010)* *2* = *3 (p* = *0.316)*

^a^Kruskal-Wallis test ; ^b^Dunn’s test.

Pairwise comparisons tests showed that only three genes *CD27* (*p* = 0.038), *MYC* (*p* = 0.027), and *POT1* (*p* = O.027) were significantly differentially expressed between the two clusters of young individuals (29.09 years and 29.31 years respectively) but with a different level of TL (2.12 and 4.35 respectively). For these three genes, a lower expression level was observed in the cluster with shorter TL (“Young Aged” cluster) as compared to the “Young” cluster (Table 2).

In order to compare these 2 youngest clusters and adjust for covariates such as Age, Sex, BMI, and tobacco, a logistic regression was performed for the 3 genes identified previously. As seen in Table 3, only *POT1* (*p* = 0.037) and BMI (*p* = 0.019) significantly differed between the two youngest clusters.

**Table 3:** Genes differentially expressed between Young Aged and Young clusters after adjustment for covariates.

Variables	Beta	SE	*p* value*
Intercept	0.645	1.581	0.639
Age	0.044	0.038	0.250
Sex	−0.374	0.472	0.428
BMI	−0.141	0.060	***0.019***
Current smoker	0.107	0.446	0.811
*CD27*	−0.310	1.810	0.864
*MYC*	1.226	2.069	0.553
*POT1*	0.676	0.325	***0.037***

*SE* Standard Error, *BMI* Body Mass Index.

^*^Logistic regression.

To investigate if these three genes were correlated to telomere length in each cluster of the youngest populations, Spearman’s correlations were performed. *POT1* was the only gene to correlate with telomere length significantly and this correlation was observed only in the “Young Aged” cluster (Table 4).

**Table 4:** Correlation between TL and genes (*CD27*, *MYC* and *POT1*) in the two clusters of young individuals.

Genes	Young (*n* = 45)		Young Aged (*n* = 68)	
	Spearman’s rho	*p* value	Spearman’s rho	*p* value
*CD27*	−0.108	0.478	0.018	0.881
*MYC*	−0.194	0.200	0.056	0.651
*POT1*	−0.046	0.764	0.357	***0.003***

## Discussion

The aim of this study was to analyze TL in a sample of 542 individuals with BD and to investigate gene expression levels of 29 age/TL-associated genes in a subgroup of 129 individuals. We first replicated the identification of a subgroup of young individuals, but with already degraded TL in an independent and larger cohort. In addition, we identified a decrease in *POT1* gene expression in the “Young Aged” group and showed that *POT1* expression level correlated with TL only in this specific subgroup.

TL is the most studied marker of aging in psychiatric diseases including BD [12]. TL is shortened in individuals with BD compared to the general population in most previous studies, but without any consensus about the potential clinical determinants [10, 12]. As expected, TL was negatively correlated with age in our sample [8].

First, we confirmed our previous finding of a subgroup of young individuals with an already pronounced telomere shortening [11] in a larger and independent sample of 542 individuals. As previously found, the two clusters of young patients—but with different TL—do not differ for all tested clinical variables. These negative results contrast with previous published ones. Shortened TL has been suggested to be associated with factors such as a high early life trauma score in both general population [32, 33] in individuals with psychiatric disorders including BD [14, 34], but also with a higher number of mood episodes [15]. However, as in our previously published study [11], we did not find an association between TL shortening and these variables in our sample. Another correlation has been suggested between long-term lithium treatment and longer TL [13, 35–37], however, the duration of lithium treatment was available for only 13% of the individuals in our database. Therefore we could not test this hypothesis. In addition, the previously observed higher anticonvulsant current use in the cluster of young patients with low TL [11] was not found in this sample. We therefore cannot conclude about the clinical determinants of accelerated cellular aging in the subgroup of young individuals with shortened TL.

Second, we analyzed the expression levels of 29 genes involved in the maintenance of TL or whose expression has been associated with age [18–20, 28, 29]. However, mRNA were available only for a subset of the individuals in the cohort, thus leading to some potential power issues. Nevertheless, expression levels of three genes (*CD27*, *MYC* and *POT1*) were significantly different between the two clusters of young individuals. After adjustments with age, sex, BMI and current smoker status only BMI and *POT1* remained significantly associated with the “Young Aged” group. The observed negative association of BMI with TL in the “Young Aged” subgroup is consistent with in a meta-analysis of 87 studies in the general population [38].

*POT1* (Protection Of Telomeres 1) encodes a protein of the “shelterin complex”. This complex includes six proteins POT1, TRF1, TRF2, TPP1 (ACD), TIN2 and RAP1 and participates in telomere maintenance [39]. POT1 directly interacts with telomeric DNA with the single-stranded G-rich tip where it prevents telomeres from degradation [39]. In addition, its affinity with telomeric DNA is enhanced by TPP1 binding [40] and the POT1-TPP1 complex has an essential role in telomere extension by recruiting telomerase to allow DNA synthesis of telomeres [41]. In this study, we found that *POT1* expression was decreased in the “Young Aged” cluster (shorter TL) as compared to the “Young” cluster suggesting that the formation of the POT1-TPP1 complex might be impaired in the “Young Aged” group. We hypothesize that this may result in less protection of the telomeric ends and a less effective recruitment of the telomerase. This is consistent with the increase telomere instability induced by *POT1* depletion in mice models [42, 43]. Furthermore, depletion of *POT1* has been shown to promote telomere fragility in several human cell lines [44–46] In addition, decreased *POT1* expression level has been associated with decreased TL in individuals with severe aplastic anemia [47], which is consistent with the correlation we observed specifically in the “Young Aged” cluster. To our knowledge, this is the first evidence of *POT1* decrease of expression in BD or any other psychiatric disorders. Moreover, we found that *POT1* expression level correlated with TL only in the “Young Aged” cluster. This suggests that, in this cluster, *POT1* expression level has a high impact on TL.

This study has several strengths and limitations. First, a large cohort of 542 individuals with BD was used for TL analyzes with a large spectrum of clinical variables available. However, some variables could not be analyzed due to missing data. This was the case especially for all somatic comorbidities variables due to the limited number of patients in each group and missing data (for example 11 subjects with diabetes versus 509 without diabetes). Indeed, many somatic comorbidities associated with an accelerated aging [48, 49] are also associated with BD. These are therefore important additional factors to analyze, but comorbidities data are often not collected properly and few studies can analyze them. Second, DNA and RNA samples were available in the same cohort, allowing us to analyze both TL and gene expression levels in the same individuals. However, RNA was not available for the whole sample and this may have led to a decrease in the power of gene expression analyses. Here, we cannot exclude some false negative results. It is also important to note that this part of the study was exploratory and carried out on a subgroup of our sample. Therefore, no correction for multiple testing was made. The aim of this study was to investigate the association between TL and expression levels of age-related genes with clinical variables in individuals with BD. However, it would have been interesting to compare *POT1* mRNA levels in healthy controls samples and individuals with BD. Finally, although we have included telomerase (*TERT*) in the tested genes, its expression level was too low and could not be analyzed. Furthermore, as blood cells were not available in this sample, telomerase activity could not be analyzed in this study. Therefore, we can’t rule out a role for telomerase (in addition to the decreased *POT1* gene expression) in the observed decrease in telomere length in the “Young Aged” group compared to the “Young” group.

In conclusion, this study confirmed the existence of a subgroup of young individuals with BD presenting with shorter TL in a larger and independent sample. None of the available clinical variables in this sample was associated with this prematurely aged phenotype. For the first time, we studied 29 genes whose expression levels are associated with age or TL in a sample of individuals with BD. We showed that a component of the shelterin complex (*POT1*) is under-expressed in the subgroup of prematurely aged patients compared to the subgroup of similar age. This downregulation is in line with previously described mechanisms of telomere instability. Therefore, our results suggest a newly described cellular mechanism in individuals with BD, that may, at least in part, contribute to telomere shortening. Further studies in independent and larger samples are required to replicate the downregulation of *POT1* and provide a better understanding of the mechanisms at stake in cellular aging in individuals with BD.

### Supplementary information

Supplementary material

## Data Availability

The RT-qPCR data that support the findings of this study are available from the corresponding author upon reasonable request.
