# Supplementary material for: Decreased telomere length in a subgroup of young individuals with bipolar disorders: replication in the FACE-BD cohort and association with the shelterin component POT1
Source: Transl Psychiatry. 2024 Mar 1;14:131. doi: 10.1038/s41398-024-02824-z (PMC10907586; doi:10.1038/s41398-024-02824-z)
Supplement: Supplementary file 1 — Supplementary material [file 41398_2024_2824_MOESM1_ESM.docx]

| **Supplementary 1. List of genes analyzed in our study.** | |
| --- | --- |
| **Genes** | **Description** |
| ACD^1^ | Shelterin Complex Subunit And Telomerase Recruitment Factor |
| ATRX^1^ | Chromatin Remodeler |
| CCDC88C^1^ | Coiled-Coil Domain Containing 88C |
| CCR7^2^ | C-C Motif Chemokine Receptor 7 |
| CD27^2^ | Molecule member of the TNF-receptor superfamily |
| CDC37^2^ | Cell Division Cycle 37, HSP90 Cochaperone |
| CHAF1A^1^ | Chromatin Assembly Factor 1 Subunit A |
| CHAF1B^1^ | Chromatin Assembly Factor 1 Subunit B |
| CRP^2^ | C-Reactive Protein |
| CX3CR1^2^ | C-X3-C Motif Chemokine Receptor 1 |
| DCLRE1B^1^ | DNA Cross-Link Repair 1B |
| DDX24^2^ | DEAD-Box Helicase 24 |
| EED^1^ | Embryonic Ectoderm Development |
| EXO1^1^ | Exonuclease 1 |
| EZH2^1^ | Enhancer Of Zeste 2 Polycomb Repressive Complex 2 Subunit |
| FGFBP2^2^ | Fibroblast Growth Factor Binding Protein 2 |
| GSK3A^2^ | Glycogen Synthase Kinase 3 Alpha |
| GZMH^2^ | Granzyme H |
| HMGB1^2^ | High Mobility Group Box 1 |
| KIAA0317^2^ | AREL1 : Apoptosis Resistant E3 Ubiquitin Protein Ligase 1 |
| LBH^2^ | LBH Regulator Of WNT Signaling Pathway |
| LDHB^2^ | Lactate Dehydrogenase B |
| LEF1^2^ | Lymphoid Enhancer Binding Factor 1 |
| LTB^2^ | Lymphotoxin Beta |
| MYC^2^ | Proto-Oncongene, BHLH Transcription Factor |
| NKG7^2^ | Natural Killer Cell Granule Protein 7 |
| PADI2^2^ | Peptidyl Arginine Deiminase 2 |
| PIK3IP1^2^ | Phosphoinositide-3-Kinase Interacting Protein 1 |
| POT1^1^ | Protection Of Telomeres 1 |
| RHOC^2^ | Ras Homolog Family Member C |
| RPS6^2^ | Ribosomal Protein S6 |
| RRM1^1^ | Ribonucleotide Reductase Catalytic Subunit M1 |
| SUZ12^1^ | Polycomb Repressive Complex 2 Subunit |
| TERF1^1^ | Telomeric Repeat Binding Factor 1 |
| TERF2^1^ | Telomeric Repeat Binding Factor 2 |
| TERF2IP^1^ | TERF2 Interacting Protein |
| TERT^1^ | Telomerase Reverse Transcriptase |
| TINF2^1^ | TERF1 Interacting Nuclear Factor 2 |
| TP53^1^ | Tumor Protein P53 |
| WRN^2^ | RecQ Like Helicase |
|  |  |
| **Reference genes** | **Description** |
| B2M | Beta-2-Microglobulin |
| HPRT1 | Hypoxanthine Phosphoribosyltransferase 1 |
| HSP90AB1 | Heat Shock Protein 90 Alpha Family Class B Member 1 |
| RPL30 | Ribosomal Protein L30 |
| RPS18 | Ribosomal Protein S18 |
| SDHA | Succinate Dehydrogenase Complex Flavoprotein Subunit A |
| ^1^ Genes related to telomere length ; ^2^ Genes related to age   \| **Variables** \| **N** \| **%** \| **Median** \| **IQR** \| \| --- \| --- \| --- \| --- \| --- \| \| Age \|  \|  \| 33 \| 15.75 \| \| Sex (female) \| 349 \| 64.4% \|  \|  \| \| BMI \|  \|  \| 24.45 \| 7.1 \| \| MADRS \|  \|  \| 8 \| 12 \| \| YMRS \|  \|  \| 1 \| 3 \| \| CSM \|  \|  \| 34 \| 11 \| \| PSQI \|  \|  \| 6 \| 5 \| \| Tobacco current smoker (yes) \| 241 \| 47.44% \|  \|  \| \| Past lifetime alcohol misuse (yes) \| 121 \| 25.74% \|  \|  \| \| Past lifetime cannabis misuse (yes) \| 118 \| 25.11% \|  \|  \| \| BD type 1 (yes) \| 251 \| 46.4% \|  \|  \| \| Age at onset \|  \|  \| 20 \| 8 \| \| Duration of BD \|  \|  \| 11.25 \| 13.03 \| \| Number of episodes \|  \|  \| 6 \| 6 \| \| Lithium (yes) \| 170 \| 39.44% \|  \|  \| \| ACA (yes) \| 217 \| 50.00% \|  \|  \| \| APA (yes) \| 185 \| 44.69% \|  \|  \| \| ATD (yes) \| 141 \| 34.06% \|  \|  \| \| Total number of medications \|  \|  \| 1.94 \| 1 \| \| CTQ total score \|  \|  \| 38 \| 17 \| \| Telomere length  BD : Bipolar Disorder ; IQR : Interquartile ; BMI : Body Mass Index ; MADRS : Montgomery Asberg Depression Rating Scale ; YMRS : Young Mania Rating Scale ; CSM : Composite Scale of Morningness ; PSQI : Pittsburgh Sleep Quality Index ; ACA : Anticonvulsant agent ; APA : Atypical Antipsychotic agent ; ATD : Antidepressant ; CTQ : Childhood Trauma Questionnaire \|  \|  \| 2.36 \| 1.71 \|   **Supplementary 2. Socio-demographic and clinical characteristics of the sample of 542 BD patients.** | |

|   **Supplementary 3. Curve plot of elbow method to determine optimized numbers of cluster (a). Cluster plot resulting from k-means clustering analysis (b).**  **Supplementary 4. Characteristics of clusters from clustering analysis on 129 patients.** | | | | | |
| --- | --- | --- | --- | --- | --- |
|  |  |  |  |  |  |
|  | **Elderly** | **Young aged** | **Young** |  |  |
| **Number of patients** | 16 | 68 | 45 |  |  |
| **Age (years), mean** | 51.38 | 29.09 | 29.31 |  |  |
| **TL, mean** | 2.61 | 2.12 | 4.35 |  |  |
